# Much more than just shyness: the impact of social anxiety disorder on educational performance across the lifespan

**DOI:** 10.1017/S0033291719003908

**Published:** 2021-04

**Authors:** Alba Vilaplana-Pérez, Ana Pérez-Vigil, Anna Sidorchuk, Gustaf Brander, Kayoko Isomura, Eva Hesselmark, Ralf Kuja-Halkola, Henrik Larsson, David Mataix-Cols, Lorena Fernández de la Cruz

**Affiliations:** 1 Department of Clinical Neuroscience, Centre for Psychiatry Research, Karolinska Institutet, Stockholm, Sweden; 2 Stockholm Health Care Services, Region Stockholm, Stockholm, Sweden; 3 Departament de Personalitat, Avaluació i Tractaments Psicològics, Universitat de València, València, Spain; 4 Department of Child and Adolescent Psychiatry and Psychology, Institute of Neuroscience, Hospital Clínic de Barcelona, Barcelona, Spain; 5 Department of Medical Epidemiology and Biostatistics, Karolinska Institutet, Stockholm, Sweden; 6 School of Medical Sciences, Örebro University, Örebro, Sweden

**Keywords:** Educational attainment, epidemiology, social anxiety disorder

## Abstract

**Background:**

Social anxiety disorder (SAD) has been linked to academic underachievement, but previous studies had methodological limitations. We investigated the association between SAD and objective indicators of educational performance, controlling for a number of covariates and unmeasured confounders shared between siblings.

**Methods:**

This population-based birth cohort study included 2 238 837 individuals born in Sweden between 1973 and 1997, followed-up until 2013. Within the cohort, 15 755 individuals had a recorded ICD-10 diagnosis of SAD in the Swedish National Patient Register. Logistic regression models tested the association between SAD and educational performance. We also identified 6488 families with full siblings discordant for SAD.

**Results:**

Compared to unexposed individuals, individuals diagnosed with SAD were less likely to pass all subjects in the last year of compulsory education [adjusted odds ratios (aOR) ranging from 0.19 to 0.44] and less likely to be eligible for a vocational or academic programme in upper secondary education [aOR = 0.31 (95% confidence interval [CI] 0.30–0.33) and aOR = 0.52 (95% CI 0.50–0.55), respectively], finish upper secondary education [aOR = 0.19 (95% CI 0.19–0.20)], start a university degree [aOR = 0.47 (95% CI 0.45–0.49)], obtain a university degree [aOR = 0.35 (95% CI 0.33–0.37)], and finish postgraduate education [aOR = 0.58 (95% CI 0.43–0.80)]. Results were attenuated but remained statistically significant in adjusted sibling comparison models. When psychiatric comorbidities were taken into account, the results were largely unchanged.

**Conclusions:**

Treatment-seeking individuals with SAD have substantially impaired academic performance throughout the formative years. Early detection and intervention are warranted to minimise the long-term socioeconomic impact of the disorder.

## Introduction

Social anxiety disorder (SAD) is characterised by a persistent fear of social or performance situations in which the person is exposed to unfamiliar people or to possible scrutiny by others, accompanied by intense anxiety and avoidance of these social situations (American Psychiatric Association, 2013). SAD is one of the most common psychiatric disorders, affecting approximately 4% of the worldwide general population – with a slightly higher prevalence in women (Ohayon & Schatzberg, 2010) – and tends to have a chronic course (Stein et al., 2017).

Similar to other disorders with frequent onset in childhood or adolescence (Lijster et al., 2017), individuals with SAD may experience difficulties in school and thus fail to meet their full educational potential (Jangmo et al., 2019; Leach & Butterworth, 2012; Van Ameringen, Mancini, & Farvolden, 2003). Indeed, previous research has reported on the association between SAD and educational impairment. The Epidemiologic Catchment Area study reported that adults with SAD (*n* = 123) or with subthreshold SAD (*n* = 248), compared to controls of the same age without the disorder (*n* = 1117), presented significant differences in three retrospectively self-reported school performance measures, including poor grades, repeating academic years, and expulsion from school (Davidson, Hughes, George, & Blazer, 1994). Likewise, the Ontario Health Survey Mental Health Supplement showed that 38.1% of a sample of 1116 individuals with SAD did not complete high school, compared to 30.1% of those without SAD (*n* = 6710), and that a lifetime diagnosis of SAD was associated with a significantly greater likelihood of having failed an academic year and leaving school before graduating from high school (Stein & Kean, 2000). Similarly, in the National Comorbidity Survey, SAD was the only early-onset psychiatric disorder that predicted failure to go on to college (Kessler, 2003). In a further study including 2128 12-to-14 year-old Swedish students who completed a self-reported screening questionnaire for social anxiety, a total of 93 probable SAD cases were identified, of which 91.4% reported school impairment due to their social fear (Gren-Landell et al., 2009). Further, in a study of 3278 15-year-old Finnish adolescents, 2070 of whom were followed-up 2 years later, a different pattern of impairment was found between socially anxious boys *v.* girls, suggesting that social anxiety may have a more deleterious effect on boys' academic functioning (Ranta, La Greca, Kaltiala-Heino, & Marttunen, 2016). By contrast, a large cross-sectional Brazilian study among university students showed that women with SAD had significantly lower grades than their female control counterparts, an effect that was not observed in men (Baptista et al., 2012).

These previous studies, while valuable, generally included small sample sizes, tended to employ cross-sectional design, used retrospective self-reported measures of educational attainment, did not control for important confounders (e.g. familial factors or comorbidities), or were exclusively focused on one specific educational milestone. In this nationwide birth cohort study, we aimed to provide more comprehensive and accurate estimates of the association of SAD with educational attainment across the lifespan. To achieve this aim, we leveraged the unique Swedish population registers, which contain detailed information about psychiatric disorders diagnosed in specialist care services, as well as prospectively collected and objectively measured educational outcomes across the person's lifespan from the entire population of Sweden. Further, in order to reduce the impact of possible confounders, we adjusted for a number of covariates, controlled for psychiatric comorbidity, and employed a sibling design to control for unmeasured familial confounders. The latter provides a stricter control of genetic and environmental factors that may have a direct influence on both the exposure and the outcomes of interest.

## Methods

All procedures contributing to this work comply with the Helsinki Declaration of 1975, as revised in 2008. The study was approved by the Stockholm Regional Ethical Review Board (reference number 2013/862-31/5). The requirement for informed consent was waived because the individuals included in this register-based study were de-identified.

### Study population and design

Using the personal identification number assigned to all Swedish residents as a key (Ludvigsson, Otterblad-Olausson, Pettersson, & Ekbom, 2009), we linked different Swedish nationwide population-based registers, including the following: (1) the Swedish Register of Total Population (Ludvigsson et al., 2016), which contains information on sociodemographic data for all individuals in Sweden since 1968; (2) the National School Register, which holds information on individual school performance collected from all municipal and independent schools from 31 December 1988 (The Swedish National Agency for Education, 2018); (3) the Longitudinal Integration Database for Health Insurance and Labour Studies (LISA, in its Swedish acronym), which annually integrates data on the labour market, education, and social sectors from all individuals living in Sweden since 1990 (Ludvigsson, Svedberg, Olén, Bruze, & Neovius, 2019); (4) the Migration Register, which provides information about migration in and out of Sweden (Ludvigsson et al., 2016); (5) the National Patient Register (NPR), which includes inpatient hospital admissions since 1964 (1973 for psychiatric information) and outpatient care since 2001 (Ludvigsson et al., 2011); (6) the Cause of Death Register, which records information on dates and causes of all deaths since 1961, with compulsory recording nationwide (Statistics Sweden, 2013a); and (7) the Multi-Generation Register, which connects individuals born in Sweden from 1932 onwards and ever registered as living in Sweden after 1960 to their biological and adoptive parents (Ekbom, 2011), and allowed us to obtain a family pedigree for each individual in our cohort.

The initial cohort consisted of all singleton births in Sweden between 1 January 1973 and 31 December 1997, totalling 2 551 071 individuals (51.4% males), who were followed-up until 31 December 2013. As described elsewhere (Pérez-Vigil et al., 2018a; Pérez-Vigil et al., 2018b), we excluded individuals with an International Classification of Diseases (ICD) diagnosis of organic brain disorder (ICD-10 codes F00–F09) and/or intellectual disabilities (ICD-8 codes 310–315, ICD-9 codes 317–319, and ICD-10 codes F70–F79) (*n* = 23 144). Likewise, we excluded individuals with two parents born outside Sweden or with missing data on the origin of the parents (*n* = 211 514) (Niederkrotenthaler et al., 2014) and those who had emigrated from Sweden (*n* = 105 566) or had died (*n* = 22 481) before the age 15 years (i.e. prior to the expected age of graduation from compulsory education) or before year 1997 (i.e. prior to introduction of ICD-10), whichever occurred last. The final study cohort comprised 2 238 837 individuals (51.3% males).

To explore the association between SAD and each of the educational milestones under study, we created separate subcohorts, which were composed of individuals who had the necessary time to achieve the corresponding educational level (and did not die or emigrate from Sweden before the age of the predicted achievement of each educational level; see ‘Statistical methods’ section).

For the sibling-comparison analyses, within each subcohort we identified a subsample of families with at least two singleton full siblings (i.e. siblings of either sex sharing the same mother and father) discordant for the diagnosis of SAD.

### Exposure

Treatment-seeking individuals with a lifetime diagnosis of SAD according to the ICD-10 definition (code F40.1) (World Health Organization, 1992), as recorded in the NPR, were considered exposed. In order to avoid misclassification, we set a minimal age threshold of 6 years for being diagnosed with the disorder. The ICD-10 code for SAD has been validated by our research group, showing good positive predictive values [PPV = 0.81 (95% confidence interval [CI] 0.72–0.88)] and substantial inter-rater reliability (*κ* = 0.72) (Vilaplana-Pérez et al., 2019). Individuals without a diagnosis of SAD were considered unexposed.

From the NPR, we also extracted all lifetime psychiatric comorbidities for the members of our cohort and organised them in five groups: (1) neuropsychiatric disorders (including pervasive developmental disorders, attention-deficit/hyperactivity disorder, Tourette syndrome and chronic tic disorder, and learning disabilities); (2) other phobic, anxiety, obsessive-compulsive, and reaction to severe stress and adjustment disorders; (3) psychotic disorders (including schizophrenia, schizotypal, and delusional disorders); (4) affective disorders (including bipolar disorder, depressive disorders, and persistent mood disorders); and (5) substance use disorders (see online Supplementary Table S1 for details on ICD codes and age thresholds).

### Outcomes

#### Compulsory education

The Swedish primary and lower secondary education are compulsory and take 9 years to complete (generally finished at ages 15–16). In this study, a subcohort of individuals graduating between 1998 and 2013 (*n* = 1 425 340) was used for this analysis. Information on individual subject grades for each participant was retrieved from the National School Register. The Swedish compulsory school system includes in total 16 compulsory subjects, for which the students are awarded the final grades upon graduation. Swedish language, English language, and mathematics are considered to be core subjects, which means that they are given extra weight in the eligibility to access upper secondary education. During the period 1998–2013, two different systems of assigning final grades were in use in Sweden and, therefore, we constructed binary variables (passed *v.* not passed) for the grades in each of 16 subjects. Furthermore, for the purposes of this study, students were also dichotomised as eligible or not eligible to access upper secondary education based on the final grades, as recorded in the National School Register. Final grades determine students' eligibility to access either vocational programmes, with a primary aim of preparing for working life, or academic programmes, which prepare for further academic studies at upper secondary school. Eligibility to access vocational programmes requires a pass grade in Swedish, English, and mathematics, and, since 2011, also requires passing five additional subjects. Academic programmes require a pass grade in Swedish, English, and mathematics, and in nine additional subjects.

#### Educational attainment after compulsory education

The database LISA was used to retrieve data on the following binary post-compulsory educational outcomes (recoded as achieved *v.* not achieved) for the full cohort (*n* = 2 238 837): finishing upper secondary education, starting a university degree, obtaining a university degree, and finishing postgraduate education (i.e. a master's or a doctoral degree).

### Statistical methods

Logistic regression models were fitted to assess the association between SAD and binary educational outcomes. For each outcome, the analysis was based on the individuals who were alive and living in Sweden at the age ‘old enough’ to start or complete a corresponding educational level. Thus, the *likelihood of passing specific subjects* and *eligibility to progress to vocational or academic upper secondary education* was assessed among individuals who graduated from compulsory education between 1998 and 2013 and did not die or emigrate from Sweden before age 15 years. An association between diagnosis of SAD and *finishing upper secondary education* was assessed in those who were born between 1973 and 1994 (i.e. aged 19 years and above at the end of follow-up in 2013) and did not die or emigrate before the age of 19 years. Analysis of *starting a university degree* included those born between 1973 and 1992 and did not die or emigrate before the age of 21 years. *Obtaining a university degree* was assessed among individuals born between 1973 and 1988 who did not die or emigrate by the age of 25 years. Finally, the likelihood of *finishing post-graduate education* was analysed in individuals born between 1973 and 1983 who did not die or emigrate before the age of 30 years. These age cut-offs are based on the mean ages to start each educational level registered by Statistics Sweden (Statistics Sweden, 2013b). First, we ran a crude model within each subcohort for the association between SAD and the corresponding educational outcomes. In a second analysis, all models were adjusted for sex, year of birth (continuous), and maternal and paternal age at the birth of each cohort member (categorised as a 5-year increment). Results were expressed as odds ratio (OR) with 95% CIs. To account for non-independence between repeated observations within families, all analyses were clustered by mother (with information on mothers being available for all study participants), with a robust sandwich estimator of standard errors (Williams, 2000).

A fixed-effects model was implemented in the subsample of full siblings discordant for SAD, where each family was considered a stratum. By design, the model controls for familial confounders shared by full siblings, including about 50% of the genetic load and unmeasured shared environmental factors, such as socioeconomic status or stable parental traits. As above, models were adjusted for sex, birth year, and parental ages. Family identification number (based on maternal and paternal identification numbers) and a robust sandwich estimator of standard errors were used to account for non-independence of observations within families (Williams, 2000).

Sensitivity analyses were performed to assess the extent to which psychiatric comorbidities influence the association between SAD and each educational milestone. To this end, the main analyses were repeated after excluding individuals with comorbid psychiatric disorders (one group of comorbid disorders at a time).

All analyses were performed using R software, version 3.4.1 (R Development Core Team, 2017), SAS, version 9.4 (SAS Institute, Cary, NC, USA) and STATA version 15.1 (StataCorp LLC, College Station, TX, USA).

## Results

### Descriptive statistics

Descriptive characteristics of the study cohort are presented in Table 1. Of the 2 238 837 individuals included, 15 755 received a diagnosis of SAD, resulting in a Kaplan–Meier estimated cumulative incidence of 1.41% (95% CI 1.38–1.44) by age 40 (online Supplementary Fig. S1). The proportion of females in the SAD cohort (8706; 55.3%) was considerably larger than in the non-exposed cohort (1 081 036; 48.7%). Those with a diagnosis of SAD were more frequently diagnosed with other psychiatric disorders, compared to those without SAD (83.1% *v.* 12.3%, respectively).

**Table 1.** Distribution of study variables among individuals born in Sweden between 1973 and 1997, with SAD and unaffected individuals from the general population

Variable	Individuals with SAD *N* = 15 755	Individuals without SAD *N* = 2 223 082
Female, *n* (%)^a^	8706 (55.3)	1 081 036 (48.3)
Age of mother at birth of individuals, mean (s.d.), years^a^	27.7 (5.4)	27.7 (5.0)
Age of father at birth of individuals, mean (s.d.), years^a^	30.7 (6.4)	30.4 (5.8)
Missing, *n* (%)	106 (0.7)	10 968 (0.5)
Comorbidity, *n* (%)		
Any	13 091 (83.1)	273 186 (12.3)
Neuropsychiatric disorders^a^^,^^b^	3938 (25)	65 019 (2.9)
Anxiety, OCD, and reaction to severe stress, and adjustment disorders^a^^,^^c^	7183 (45.6)	50 746 (2.3)
Psychotic disorders^a^^,^^d^	905 (5.7)	12 008 (0.5)
Affective disorders^a^^,^^e^	9359 (59.4)	113 941 (5.1)
Substance use disorders^a^	3760 (24)	92 046 (4.1)

OCD, obsessive-compulsive disorder; s.d., standard deviation.

a Statistically significant between-group difference (*p* < 0.001) determined with a χ^2^ test or an independent samples, two-tailed *t* test.

b Includes pervasive developmental disorders, attention-deficit/hyperactivity disorder, Tourette's and chronic tic disorders and learning disabilities.

c Excludes social anxiety disorder.

d Includes schizophrenia, schizotypal, and delusional disorders.

e Includes bipolar disorder, depressive disorders, and persistent mood disorder.

### Compulsory education

Analyses of the specific school subjects revealed that individuals with a diagnosis of SAD were significantly less likely to pass all subjects in the last year of compulsory education [adjusted OR (aOR) ranging from 0.19 to 0.44]. For example, individuals with SAD had 67%, 56%, and 67% lower odds of passing each of the core subjects (Swedish, English, and mathematics, respectively) (online Supplementary Table S2). The results of the sibling comparison models resulted in attenuated estimates, but the results were in the same direction (aORs ranging from 0.34 to 0.66) (online Supplementary Table S3).

Individuals with SAD were significantly less likely to be eligible to access a vocational or an academic programme in upper secondary education, compared to the general population [77.3% in the exposed group were eligible for a vocational programme, compared to 91.4% in the unexposed group, aOR = 0.31 (95% CI 0.30–0.33); and 46.4% in the exposed group were eligible for an academic programme, compared to 62.7% in the unexposed group, aOR = 0.52 (95% CI 0.50–0.55)]. There were no significant sex differences in eligibility for an academic programme in the exposed group, but females with SAD were significantly less likely to be eligible for a vocational programme, compared to males with SAD [aOR for females = 0.28 (95% CI 0.26–0.30) *v.* aOR for males = 0.36 (95% CI 0.33–0.38)] (Table 2).

**Table 2.** Odds ratios and corresponding 95% CIs for educational attainment among individuals with lifetime SAD, compared with unaffected individuals from the general population, stratified by gender

	Individuals with SAD	Individuals without SAD	Unadjusted model^a^	Adjusted model^b^
	*n* (%)	*n* (%)	OR (95% CI)	OR (95% CI)
*Compulsory education, eligibility to access upper secondary education*^c^	***n* = 10 093**	***n* = 1 415 247**		
Vocational programme				
All	7803 (77.31)	1 292 842 (91.35)	**0.32 (0.31–0.34)**	**0.31 (0.30–0.33)**
Female	4543 (78.05)	638 671 (92.61)	**0.28 (0.27–0.30)**	**0.28 (0.26–0.30)**
Male	3260 (76.31)	654 171 (90.16)	**0.35 (0.33–0.38)**	**0.36 (0.33–0.38)**
Academic programme				
All	4678 (46.35)	887 028 (62.68)	**0.51 (0.49–0.54)**	**0.52 (0.50–0.55)**
Female	2739 (47.05)	439 525 (63.73)	**0.51 (0.48–0.53)**	**0.52 (0.49–0.54)**
Male	1939 (45.39)	447 503 (61.67)	**0.52 (0.49–0.55)**	**0.54 (0.50–0.57)**
*Post-compulsory education*				
Finishing upper secondary education^d^	***n* = 14 997**	***n* = 1 983 974**		
All	6878 (45.86)	1 602 207 (80.76)	**0.20 (0.20–0.21)**	**0.19 (0.19–0.20)**
Female	3875 (47.15)	800 591 (83.04)	**0.18 (0.17–0.19)**	**0.18 (0.17–0.19)**
Male	3003 (44.30)	801 616 (78.60)	**0.22 (0.21–0.23)**	**0.21 (0.20–0.22)**
Starting a university degree^e^	***n* = 13 901**	***n* = 1 781 080**		
All	3226 (23.21)	685 144 **(**38.46)	**0.48 (0.46–0.50)**	**0.47 (0.45–0.49**)
Female	1962 (26.05)	394 855 (45.78)	**0.42 (0.40–0.44)**	**0.43 (0.40–0.45)**
Male	1264 (19.85)	290 289 (31.60)	**0.54 (0.50–0.57)**	**0.54 (0.51–0.58)**
Obtaining a university degree^f^	***n* = 10 731**	***n* = 1 346 110**		
All	1187 (11.06)	350 069 (26.01)	**0.35 (0.33–0.38)**	**0.35 (0.33–0.37)**
Female	778 (13.74)	219 742 (33.80)	**0.31 (0.29–0.34)**	**0.33 (0.30–0.35)**
Male	409 (8.07)	130 327 (18.73)	**0.38 (0.34–0.42)**	**0.40 (0.36–0.45)**
Finishing post-graduate education^g^	***n* = 6680**	***n* = 892 645**		
All	40 (0.60)	10 181 (1.14)	**0.52 (0.38–0.71)**	**0.58 (0.43–0.80)**
Female	19 (0.55)	4643 (1.08)	**0.51 (0.33–0.80)**	**0.57 (0.36–0.90)**
Male	21 (0.64)	5538 (1.20)	**0.54 (0.35–0.82)**	**0.60 (0.39–0.92)**

CI, confidence interval; OR, odds ratio; SAD, social anxiety disorder.

*Note*: Statistically significant findings are highlighted in bold.

a Crude logistic regression model clustered by mother with robust standard error estimation (sandwich estimator).

b Multivariate logistic regression model adjusted for sex, year of birth, maternal age at birth and paternal age at birth, and clustered by mother with a robust standard error estimation (sandwich estimator). Separate analyses for males and females did not adjust for sex.

c Subcohort of individuals graduating compulsory school between 1998 and 2013 and not having died or emigrated from Sweden before age of 15 years.

d Subcohort of individuals born in 1973–1994 and not having died or emigrated from Sweden before age of 19 years.

e Subcohort of individuals born in 1973–1992 and not having died or emigrated from Sweden before age of 21 years.

f Subcohort of individuals born in 1973–1988 and not having died or emigrated from Sweden before age of 25 years.

g Subcohort of individuals born in 1973–1983 and not having died or emigrated from Sweden before age of 30 years.

Similar results were obtained in the sibling comparison models, but with attenuated estimates [aOR for vocational programme = 0.53 (95% CI 0.48–0.59); aOR for academic programme = 0.63 (95% CI 0.58–0.69)] (Table 3).

**Table 3.** Odds ratios and corresponding 95% CIs for educational attainment among individuals with lifetime SAD, compared with their unaffected full siblings

	Full siblings with SAD	Full siblings without SAD	Unadjusted model^a^	Adjusted model^b^
	*n* (%)^c^	*n* (%)^d^	OR (95% CI)	OR (95% CI)
*Compulsory education, eligibility to access upper secondary education*^e^	***n* = 6488**	***n* = 9024**		
Vocational programme	5165 (79.61)	7706 (85.39)	**0.56 (0.51–0.62)**	**0.53 (0.48–0.59)**
Academic programme	3099 (47.77)	4929 (54.62)	**0.65 (0.59–0.70)**	**0.63 (0.58–0.69)**
*Post-compulsory education*				
Finishing upper secondary education^f^	***n* = 10 153**	***n* = 14 981**		
	4802 (47.30)	10 171 (67.89)	**0.31 (0.29–0.34)**	**0.31 (0.29–0.33)**
Starting a university degree^g^	***n* = 9121**	***n* = 13 247**		
	2313 (25.26)	4410 (33.29)	**0.55 (0.51–0.59)**	**0.54 (0.50–0.59**)
Obtaining a university degree^h^	***n* = 6471**	***n* = 8765**		
	820 (12.67)	1806 (20.60)	**0.48 (0.43–0.53)**	**0.50 (0.45–0.56)**
Finishing post graduate education^i^	***n* = 3352**	***n* = 4059**		
	23 (0.69)	39 (0.96)	0.66 (0.39–1.10)	NA

CI, confidence interval; NA, not applicable due to underpowered analysis; OR, odds ratio; SAD, social anxiety disorder.

*Note*: Statistically significant findings are highlighted in bold. Siblings retrieved from the same subcohort, corresponding to each educational milestone, and fulfil the same inclusion/exclusion criteria.

a Crude fixed-effect (i.e. conditional) logistic regression model where each family considered a stratum, with robust standard error estimation (sandwich estimator).

b Multivariate fixed-effect (i.e. conditional) logistic regression model, where each family considered a stratum, adjusted for sex, year of birth, maternal age at birth and paternal age at birth, with a robust standard error estimation (sandwich estimator).

c Individuals with SAD who have at least one full sibling without SAD; the analysis includes only full sibling pairs who are discordant be both exposure and outcome.

d Individuals without SAD who have at least one full sibling with SAD; the analysis includes only full sibling pairs who are discordant be both exposure and outcome.

e Subcohort of individuals graduating compulsory school between 1998 and 2013 and not having died or emigrated from Sweden before age of 15 years.

f Subcohort of individuals born in 1973–1994 and not having died or emigrated from Sweden before age of 19 years.

g Subcohort of individuals born in 1973–1992 and not having died or emigrated from Sweden before age of 21 years.

h Subcohort of individuals born in 1973–1988 and not having died or emigrated from Sweden before age of 25 years.

i Subcohort of individuals born in 1973–1983 and not having died or emigrated from Sweden before age of 30 years.

### Educational attainment after compulsory education

Individuals with SAD were significantly less likely to achieve each of the assessed post-compulsory educational milestones during the study period, compared to the individuals without SAD. Specifically, in the adjusted models, SAD cases had 81% lower odds of finishing upper secondary education [aOR = 0.19 (95% CI 0.19–0.20)], 53% lower odds of starting a university degree [aOR = 0.47 (95% CI 0.45–0.49)], 65% lower odds of obtaining a university degree [aOR = 0.35 (95% CI 0.33–0.37)], and 42% lower odds of achieving a post-graduate education [aOR = 0.58 (95% CI 0.43–0.80)] (Table 2). Regarding sex differences, females were significantly more impaired than males across all educational levels, except for finishing post-graduate education. The educational level presenting the largest sex difference was starting a university degree [aOR for females = 0.43 (95% CI 0.40–0.45) *v.* aOR for males = 0.54 (95% CI 0.51–0.58)].

Estimates in the sibling comparison models were again attenuated, although individuals with SAD still showed more impairment, compared to their unaffected siblings (Table 3).

### Sensitivity analysis

The exclusion of individuals with different groups of comorbidities resulted in estimates similar to the ones in the main analysis (Table 4). An underpowered analysis of the impact of comorbid disorders on the likelihood of finishing post-graduate education precluded us from drawing definite conclusions. However, the observed measures of associations for the last educational milestone (although not all reaching significance) suggested the individuals with SAD to be less likely to achieve such educational outcome, regardless of comorbid disorders, thus, supporting the results from the main analysis.

**Table 4.** Adjusted odds ratios and corresponding 95% CIs for educational attainment among individuals with lifetime SAD, compared with unaffected individuals from the general population, excluding different groups of comorbidities and stratified by gender

	Whole cohort	Excluding neuropsychiatric disorders	Excluding anxiety disorders	Excluding psychotic disorders	Excluding affective disorders	Excluding substance use disorders
*Compulsory education, eligibility to access upper secondary education*^a^						
Vocational programme						
All	**0.31 (0.30–0.33)**	**0.35 (0.33–0.38)**	**0.32 (0.30–0.34)**	**0.31 (0.30–0.33)**	**0.26 (0.25–0.28)**	**0.32 (0.30–0.33)**
Female	**0.28 (0.26–0.30)**	**0.32 (0.29–0.34)**	**0.28 (0.25–0.30)**	**0.28 (0.26–0.30)**	**0.24 (0.21–0.26)**	**0.29 (0.27–0.31)**
Male	**0.36 (0.33–0.38)**	**0.41 (0.37–0.45)**	**0.36 (0.33–0.40)**	**0.35 (0.33–0.38)**	**0.29 (0.26–0.32)**	**0.35 (0.32–0.38)**
Academic programme						
All	**0.52 (0.50–0.55)**	**0.59 (0.56–0.62)**	**0.54 (0.52–0.57)**	**0.53 (0.51–0.55)**	**0.50 (0.47–0.54)**	**0.54 (0.52–0.57)**
Female	**0.52 (0.49–0.54)**	**0.58 (0.54–0.61)**	**0.52 (0.49–0.56)**	**0.52 (0.49–0.55)**	**0.52 (0.48–0.57)**	**0.54 (0.51–0.57)**
Male	**0.54 (0.50–0.57)**	**0.61 (0.57–0.66)**	**0.57 (0.53–0.62)**	**0.54 (0.51–0.58)**	**0.48 (0.44–0.53)**	**0.55 (0.52–0.59)**
*Post-compulsory education*						
Finishing upper secondary education^b^						
All	**0.19 (0.19–0.20)**	**0.22 (0.21–0.23)**	**0.21 (0.20–0.22)**	**0.20 (0.19–0.20)**	**0.20 (0.19–0.21)**	**0.21 (0.20–0.22)**
Female	**0.18 (0.17–0.19)**	**0.20 (0.19–0.21)**	**0.19 (0.18–0.20)**	**0.18 (0.17–0.19)**	**0.18 (0.17–0.20)**	**0.20 (0.19–0.21)**
Male	**0.21 (0.20–0.22)**	**0.24 (0.23–0.26)**	**0.23 (0.21–0.24)**	**0.22 (0.21–0.23)**	**0.21 (0.19–0.22)**	**0.23 (0.22–0.24)**
Starting a university degree^c^						
All	**0.47 (0.45–0.49**)	**0.54 (0.52–0.56)**	**0.56 (0.53–0.59)**	**0.48 (0.46–0.50)**	**0.49 (0.46–0.52)**	**0.54 (0.52–0.57)**
Female	**0.43 (0.40–0.45)**	**0.49 (0.46–0.52)**	**0.51 (0.48–0.55)**	**0.43 (0.41–0.45)**	**0.45 (0.41–0.49)**	**0.49 (0.45–0.51)**
Male	**0.54 (0.51–0.58)**	**0.64 (0.59–0.68)**	**0.62 (0.57–0.67)**	**0.56 (0.52–0.60)**	**0.55 (0.50–0.60)**	**0.67 (0.62–0.71)**
Obtaining a university degree^d^						
All	**0.35 (0.33–0.37)**	**0.41 (0.38–0.43)**	**0.41 (0.38–0.45)**	**0.36 (0.34–0.38)**	**0.42 (0.38–0.46)**	**0.41 (0.39–0.44)**
Female	**0.33 (0.30–0.35)**	**0.37 (0.34–0.40)**	**0.39 (0.35–0.43)**	**0.33 (0.31–0.36)**	**0.39 (0.34–0.43)**	**0.37 (0.35–0.41)**
Male	**0.40 (0.36–0.45)**	**0.50 (0.45–0.55)**	**0.46 (0.40–0.52)**	**0.42 (0.38–0.47)**	**0.47 (0.41–0.54)**	**0.51 (0.45–0.57)**
Finishing post graduate education^e^						
All	**0.58 (0.43–0.80)**	**0.63 (0.45–0.88)**	0.75 (0.51–1.10)	**0.56 (0.41–0.78)**	**0.60 (0.37–0.97)**	0.75 (0.55–1.03)
Female	**0.57 (0.36–0.90)**	0.64 (0.40–1.02)	0.69 (0.38–1.25)	**0.53 (0.33–0.86)**	0.64 (0.32–1.28)	0.69 (0.44–1.09)
Male	**0.60 (0.39–0.92)**	**0.62 (0.38–0.99)**	0.79 (0.48–1.32)	**0.59 (0.38–0.93)**	0.57 (0.30–1.11)	0.81 (0.52–1.27)

*Note:* All odds ratios are obtained by multivariate logistic regression model adjusted for sex, year of birth, maternal age at birth and paternal age at birth and clustered by mother with robust standard error estimation (sandwich estimator). Separate analyses for males and females did not adjust for sex. Statistically significant findings are highlighted in bold. ICD codes for the disorders that constitute each group of psychiatric comorbidities are reported in online Supplementary Table S1.

a Subcohort of individuals graduating compulsory school between 1998 and 2013 and not having died or emigrated from Sweden before age of 15 years.

b Subcohort of individuals born in 1973–1994 and not having died or emigrated from Sweden before age of 19 years.

c Subcohort of individuals born in 1973–1992 and not having died or emigrated from Sweden before age of 21 years.

d Subcohort of individuals born in 1973–1988 and not having died or emigrated from Sweden before age of 25 years.

e Subcohort of individuals born in 1973–1983 and not having died or emigrated from Sweden before age of 30 years.

## Discussion

In this nationwide study, we analysed data from a large cohort of more than 2 million individuals from the Swedish population and demonstrated that individuals diagnosed with SAD are much more likely to underachieve across all levels of education. These results confirm and expand previous research, mainly based on modest sample sizes and self-reported outcomes (Davidson et al., 1994; Gren-Landell et al., 2009; Kessler, 2003; Lijster et al., 2018; Ohayon & Schatzberg, 2010; Ranta et al., 2016; Stein & Kean, 2000), by including objectively measured educational outcomes, adopting a lifespan perspective, and using a sibling comparison to control for unmeasured genetic and environmental factors that are shared by siblings.

Individuals with SAD were less likely to pass all subjects in compulsory education and were also less likely to achieve all the educational milestones under study, from compulsory education to post-graduate education, compared to individuals from the general population and also to their unaffected full siblings. The educational level that showed the greatest impairment was finishing upper secondary education; individuals with SAD had 81% lower odds of achieving this milestone. These results are in line with previously described difficulties of young people with SAD in their transition from high school to university (Kessler, 2003).

Despite the fact that more women than men, regardless of their exposure status, accessed all education levels (except for post-graduate education), women with SAD had lower odds of achieving most educational milestones, compared to men with SAD, particularly starting and obtaining a university degree. Our results are in line with those reported by Baptista et al. (2012) showing that female university students with SAD had significantly lower grades than those without SAD, which did not occur when male students with SAD were compared to those without SAD (Baptista et al., 2012). Collectively, these findings suggest that women with SAD may be particularly vulnerable to scholastic underachievement.

The discordant sibling comparison resulted in attenuated estimates, indicating that part of the observed associations was explained by factors shared by siblings, including genetic and shared familial factors. Indeed, socioeconomic status, parental psychopathology, and the educational level of the parents have been previously associated with school performance in the offspring in their own right (Esch et al., 2014; Schlechter & Milevsky, 2010). Similarly, it is well known that educational attainment is heritable to a high degree (Rimfeld et al., 2018; Shakeshaft et al., 2013). However, these factors shared between siblings did not explain the whole association since SAD-affected individuals were still substantially impaired across all educational levels compared to their unaffected siblings. Similarly, systematically removing various groups of psychiatric comorbidities from our analysis did not substantially alter the results. Taken together, these results strongly suggest that SAD is associated with profound impairments in educational performance in its own right.

Given the robust associations between an individual's educational level and a wide range of health and socioeconomic outcomes later in life (Braveman & Gottlieb, 2014; OECD, 2017) our results indicate that early detection and treatment of SAD should be prioritised. Whether improvement in SAD symptoms or remission from the disorder has a positive effect on educational outcomes is unknown, although it seems plausible. However, despite the existence of evidence-based treatments for the disorder (Pilling et al., 2013), only about half of individuals with SAD ever seek treatment and, when they do, it is often 10–15 years after symptom onset (Grant et al., 2005; Masia Warner et al., 2005). Moreover, parents commonly underestimate the extent of adversity experienced by young people with SAD (Ginsburg, Siqueland, Masia-Warner, & Hedtke, 2004; Masia, Klein, Storch, & Corda, 2001); anxiety symptoms are sometimes not easily observable and dismissed as just ‘shyness’ (Wessely, 2008). While our study is based on treatment-seeking individuals seen in specialist care, and the results may not generalise to milder forms of the disorder, they clearly indicate that SAD is much more than just shyness and can have far-reaching consequences for the individual.

Educating school staff and peers in the recognition of SAD symptoms may be a way of contributing to the early detection of SAD and facilitating referrals to relevant mental health services (Masia Warner et al., 2005, 2016; Masia Warner, Fisher, Shrout, Rathor, & Klein, 2007). For example, a two-step screening programme of social anxiety symptoms, including self-report measures and a brief phone interview with parents, proved to be effective at detecting SAD cases in a high school context (Sweeney et al., 2015). Furthermore, some clinical intervention programmes for SAD have been adapted to school contexts in order to help young people with SAD to progress in their educational goals. For instance, the Social Effectiveness Therapy for Children (SET-C) programme (Beidel, Turner, & Morris, 2000) was adapted into the 12-week Skills for Academic and Social Success (SASS) programme (Masia Warner, Colognori, & Lynch, 2018), which was shown to be superior than a non-specific counselling in reducing social anxiety and increasing school functioning in 138 high school students (Masia Warner et al., 2016). School-based interventions for SAD may have several advantages over individual treatments as they have demonstrated larger effect sizes in a recent meta-analysis (Scaini, Belotti, Ogliari, & Battaglia, 2016), in part because the school context allows for naturalistic exposures, real-world practice, and promotion of skills generalisation and better environment (Sweeney et al., 2015). The use of new technologies may also facilitate broader access to cognitive-behaviour therapy, which is efficacious but seldom available for young people and adults with SAD (Hedman et al., 2011; Nordh et al., 2017).

Strengths of the current study include the large population-based cohort with objective educational outcome data collected prospectively from nationwide administrative records, which ensured minimal risk of selection, social desirability, and recall biases. Additionally, the validity and reliability of the Swedish ICD-10 code for SAD is good (Vilaplana-Pérez et al., 2019). The follow-up period of over 20 years allowed sufficient time for the various cohorts to reach the relevant educational milestones, including post-graduate studies. The discordant sibling design provided unprecedented control of unmeasured confounders shared by full siblings. However, the study also has limitations. The study is based on treatment-seeking individuals diagnosed by specialists, which may affect the generalisability of the findings to non-treatment seeking persons or individuals diagnosed by general practitioners or non-medical professionals (e.g. psychologists). Further, outpatient records are only available from 2001, and as the disorder does not usually require hospitalisation, the vast majority of diagnosed SAD cases in our cohort were collected from 2001 onwards. An additional limitation is that the NPR does not include measures of symptom severity, which may have a clear association with the eventual educational attainment of an individual.

## Conclusion

Treatment-seeking individuals with SAD, particularly females, have substantially impaired academic performance throughout the formative years. Early detection and intervention are warranted to minimise the long-term socioeconomic impact of the disorder.

## Supplementary material

For supplementary material accompanying this paper visit https://doi.org/10.1017/S0033291719003908. click here to view supplementary material
