# Supplementary material for: Much more than just shyness: the impact of social anxiety disorder on educational performance across the lifespan
Source: Psychol Med. 2020 Jan 7;51(5):861–9. doi: 10.1017/S0033291719003908 (PMC8108394; doi:10.1017/S0033291719003908)
Supplement: Supplementary file 1 [file S0033291719003908sup.zip › S0033291719003908sup002.docx]

**SUPPLEMENTARY MATERIAL.**

**Supplementary Table 1.** Comorbidities taken into account in the study: diagnostic groups with ICD codes and age thresholds.

| **Diagnostic groups** | **ICD-10** | **Minimal age of diagnosis** |
| --- | --- | --- |
| **1. Neuropsychiatric disorders** |  |  |
| Pervasive developmental disorders, attention-deficit/hyperactivity disorder, learning disabilities, Tourette syndrome/chronic tic disorder | F81, F84, F90, F95 | ≥ 3-year-old  (except for pervasive developmental disorders ≥1 year old) |
| 1. **Anxiety, obsessive-compulsive and reaction**   **to severe stress and adjustment disorders** | | |
| Anxiety disorders, obsessive-compulsive disorder, reaction to severe stress, adjustment disorders | F40-F43  (excluding F40.1, corresponding to SAD) | ≥6 years old |
| 1. **Psychotic disorders** |  |  |
| Schizophrenia, schizotypal disorder, delusional disorder | F20-F29, F32.3,  x.5 in F10-F19 | ≥10 years old |
| 1. **Affective disorders** |  |  |
| Bipolar disorder, depressive disorder, persistent mood disorder | F30-F39  except 32.3 | ≥6 years old |
| 1. **Substance use disorders** |  |  |
|  | F10-F19,  except for x.5 and F17 | ≥10 years old |

**Supplementary Table 2.** Odds ratios (OR) and corresponding 95% confidence intervals (CI) for passing specific subjects at the end of compulsory education among individuals with social anxiety disorder (SAD), compared with unaffected individuals from the general population. Data from the subcohort of individuals graduating compulsory school between 1998 and 2013.

|  | **Individuals with SAD**  **(n=10,093)** | **Individuals without SAD**  **(n=1,415,247)** | **Unadjusted model^a^** | **Adjusted^b^** |
| --- | --- | --- | --- | --- |
|  | **n (%)** | **n (%)** | **OR (95% CI)** | **OR (95% CI)** |
| **Core subjects** |  |  |  |  |
| Swedish language | 8,652 (85.72) | 1,351,134 (95.47) | **0.35 (0.32-0.37)** | **0.33 (0.31-0.36)** |
| *missing* | 475 (4.71) | 11,772 (0.83) |  |  |
| English language | 8,663 (85.83) | 1,339,149 (94.62) | **0.44 (0.41-0.47)** | **0.44 (0.41-0.47)** |
| *missing* | 469 (4.65) | 10,242 (0.72) |  |  |
| Mathematics | 8,125 (80.50) | 1,322,233 (93.43) | **0.34 (0.32-0.36)** | **0.33 (0.31-0.35)** |
| *Missing* | 470 (4.66) | 10,228 (0.72) |  |  |
| **Additional subjects** |  |  |  |  |
| Arts | 8,303 (82.26) | 1,351,626 (95.50) | **0.25 (0.23-0.26)** | **0.24 (0.23-0.26)** |
| *missing* | 472 (4.68) | 10,623 (0.75) |  |  |
| Biology | 6,316 (62.58) | 1,099,040 (77.66) | **0.29 (0.28-0.31)** | **0.29 (0.27-0.31)** |
| *missing* | 2,123 (21.03) | 231,399 (16.35) |  |  |
| Chemistry | 5,945 (58.90) | 1,076,595 (76.07) | **0.29 (0.28-0.31)** | **0.29 (0.27-0.30)** |
| *missing* | 2,128 (21.08) | 232,008 (16.39) |  |  |
| Geography | 5,482 (54.31) | 942,739 (66.61) | **0.29 (0.27-0.31)** | **0.29 (0.27-0.31)** |
| *missing* | 3,354 (33.32) | 409,932 (28.97) |  |  |
| Handcraft textile/wood | 8,263 (81.87) | 1,358,371 (95.98) | **0.21 (0.19-0.22)** | **0.21 (0.19-0.22)** |
| *missing* | 483 (4.79) | 11,141 (0.79) |  |  |
| History | 5,489 (54.38) | 941,373 (66.52) | **0.30 (0.28-0.32)** | **0.30 (0.28-0.32)** |
| *missing* | 3,351 (33.20) | 409,891 (28.96) |  |  |
| Home and consumer studies | 6,218 (61.61) | 1,120,195 (79.15) | **0.21 (0.20-0.22)** | **0.21 (0.20-0.22)** |
| *missing* | 2,651 (26.27) | 249,000 (17.59) |  |  |
| Knowledge of society | 5,502 (54.51) | 944,176 (66.71) | **0.29 (0.27-0.31)** | **0.29 (0.27-0.31)** |
| *missing* | 3,355 (33.24) | 409,886 (28.96) |  |  |
| Music | 8,051 (79.77) | 1,342,094 (94.83) | **0.24 (0.23-0.25)** | **0.24 (0.22-0.25)** |
| *missing* | 484 (4.80) | 10,825 (0.76) |  |  |
| Physics | 5,979 (59.24) | 1,083,126 (76.53) | **0.28 (0.26-0.29)** | **0.28 (0.26-0.29)** |
| *missing* | 2,125 (21.05) | 231,940 (16.39) |  |  |
| Religion | 5,502 (54.51) | 943,139 (66.64) | **0.29 (0.28-0.31)** | **0.29 (0.27-0.31)** |
| *missing* | 3,356 (33.25) | 410,020 (28.97) |  |  |
| Sports and health | 6,919 (68.55) | 1,310,422 (92.59) | **0.18 (0.17-0.19)** | **0.19 (0.18-0.20)** |
| *missing* | 489 (4.84) | 11,140 (0.79) |  |  |
| Technology | 7,827 (77.55) | 1,332,625 (94.16) | **0.24 (0.22-0.25)** | **0.24 (0.23-0.25)** |
| *missing* | 487 (4.83) | 11,048 (0.78) |  |  |

*Note:* Statistically significant findings are highlighted in bold. Siblings retrieved from the same subcohort and fulfill the same inclusion/exclusion criteria

^a^ Crude logistic regression model clustered by mother with robust standard error estimation (sandwich estimator)

^b^ Multivariate logistic regression model adjusted for sex, year of birth, maternal age at birth and paternal age at birth, and clustered by mother with a robust standard error estimation (sandwich estimator)

*Abbreviations*: CI confidence interval; OR odds ratio; SAD social anxiety disorder.

**Supplementary Table 3.** Odds ratios (OR) and corresponding 95% confidence intervals (CI) for passing specific subjects at the end of compulsory education among individuals with social anxiety disorder (SAD), compared with their unaffected full siblings. Data from the subcohort of individuals graduating compulsory school between 1998 and 2013.

|  | **Full siblings with SAD**  **(n=6,488)^a^** | **Full siblings without SAD**  **(n=9,024)^b^** | **Full siblings unadjusted^c^** | **Full siblings adjusted^d^** |
| --- | --- | --- | --- | --- |
|  | **n (%)** | **n (%)** | **OR (95% CI)** | **OR (95% CI)** |
| **Core subjects** |  |  |  |  |
| Swedish language | 5,667 (87.35) | 8,266 (91.60) | **0.63 (0.55-0.73)** | **0.57 (0.49-0.66)** |
| *missing* | 273 (4.21) | 179 (1.98) |  |  |
| English language | 5,662 (87.27) | 8,206 (90.94) | **0.69 (0.60-0.80)** | **0.66 (0.57-0.76)** |
| *missing* | 269 (4.15) | 174 (1.93) |  |  |
| Mathematics | 5,377 (82.88) | 7,930 (87.88) | **0.65 (0.58-0.73)** | **0.63 (0.55-0.71)** |
| *missing* | 268 (4.13) | 174 (1.93) |  |  |
| **Additional subjects** |  |  |  |  |
| Arts | 5,438 (83.82) | 8,156 (90.38) | **0.50 (0.44-0.57)** | **0.45 (0.39-0.52)** |
| *missing* | 271 (4.18) | 177 (1.96) |  |  |
| Biology | 4,172 (64.30) | 6,365 (70.53) | **0.54 (0.47-0.61)** | **0.50 (0.44-0.57)** |
| *missing* | 1,354 (20.87) | 1,709 (18.94) |  |  |
| Chemistry | 3,930 (60.57) | 6,129 (67.92) | **0.53 (0.47-0.59)** | **0.50 (0.44-0.56)** |
| *missing* | 1,358 (20.93) | 1,715 (19.00) |  |  |
| Geography | 3,570 (55.02) | 5,418 (60.04) | **0.56 (0.49-0.65)** | **0.53 (0.46-0.62)** |
| *missing* | 2,196 (33.85) | 2,869 (31.79) |  |  |
| Handcraft textile/wood | 5,411 (83.40) | 8,234 (91.25) | **0.41 (0.36-0.46)** | **0.38 (0.34-0.44)** |
| *missing* | 278 (4.28) | 180 (1.99) |  |  |
| History | 3,560 (54.87) | 5,451 (60.41) | **0.53 (0.46-0.62)** | **0.52 (0.45-0.60)** |
| *missing* | 2,195 (33.83) | 2,870 (31.80) |  |  |
| Home and consumer studies | 4,398 (67.79) | 6,708 (74.34) | **0.43 (0.38-0.50)** | **0.40 (0.34-0.46)** |
| *missing* | 1,310 (20.19) | 1,680 (18.62) |  |  |
| Knowledge of society | 3,582 (55.21) | 5,468 (60.59) | **0.54 (0.46-0.62)** | **0.52 (0.44-0.60)** |
| *missing* | 2,198 (33.88) | 2,867 (31.77) |  |  |
| Music | 5,277 (81.33) | 8,059 (89.31) | **0.45 (0.40-0.51)** | **0.42 (0.37-0.47)** |
| *missing* | 275 (4.24) | 180 (1.99) |  |  |
| Physics | 3,961 (61.05) | 6,191 (68.61) | **0.50 (0.44-0.56)** | **0.47 (0.42-0.53)** |
| *missing* | 1,356 (20.90) | 1,714 (18.99) |  |  |
| Religion | 3,576 (55.12) | 5,460 (60.51) | **0.53 (0.46-0.61)** | **0.48 (0.41-0.57)** |
| *missing* | 2,198 (33.88) | 2,870 (31.80) |  |  |
| Sports and health | 4,567 (70.39) | 7,566 (83.84) | **0.34 (0.31-0.38)** | **0.34 (0.31-0.38)** |
| *missing* | 278 (4.28) | 184 (2.04) |  |  |
| Technology | 5,178 (79.81) | 7,935 (87.93) | **0.47 (0.42-0.52)** | **0.45 (0.40-0.51)** |
| *missing* | 278 (4.28) | 183 (2.03) |  |  |

*Note:* Statistically significant findings are highlighted in bold. Siblings retrieved from the same subcohort and fulfill the same inclusion/exclusion criteria

^a^ Individuals with SAD who have at least one full sibling without SAD; the analysis includes only full sibling pairs who are discordant be both exposure and outcome

^b^ Individuals without SAD who have at least one full sibling with SAD; the analysis includes only full sibling pairs who are discordant be both exposure and outcome

^c^ Crude fixed-effect (i.e., conditional) logistic regression model where each family considered a stratum, with robust standard error estimation (sandwich estimator)

^d^ Multivariate fixed-effect (i.e., conditional) logistic regression model, where each family considered a stratum, adjusted for sex, year of birth, maternal age at birth and paternal age at birth, with a robust standard error estimation (sandwich estimator)

*Abbreviations*: CI confidence interval; OR odds ratio; SAD social anxiety disorder.
