# Supplementary figures and images for: Much more than just shyness: the impact of social anxiety disorder on educational performance across the lifespan
Source: Psychol Med. 2020 Jan 7;51(5):861–9. doi: 10.1017/S0033291719003908 (PMC8108394; doi:10.1017/S0033291719003908)

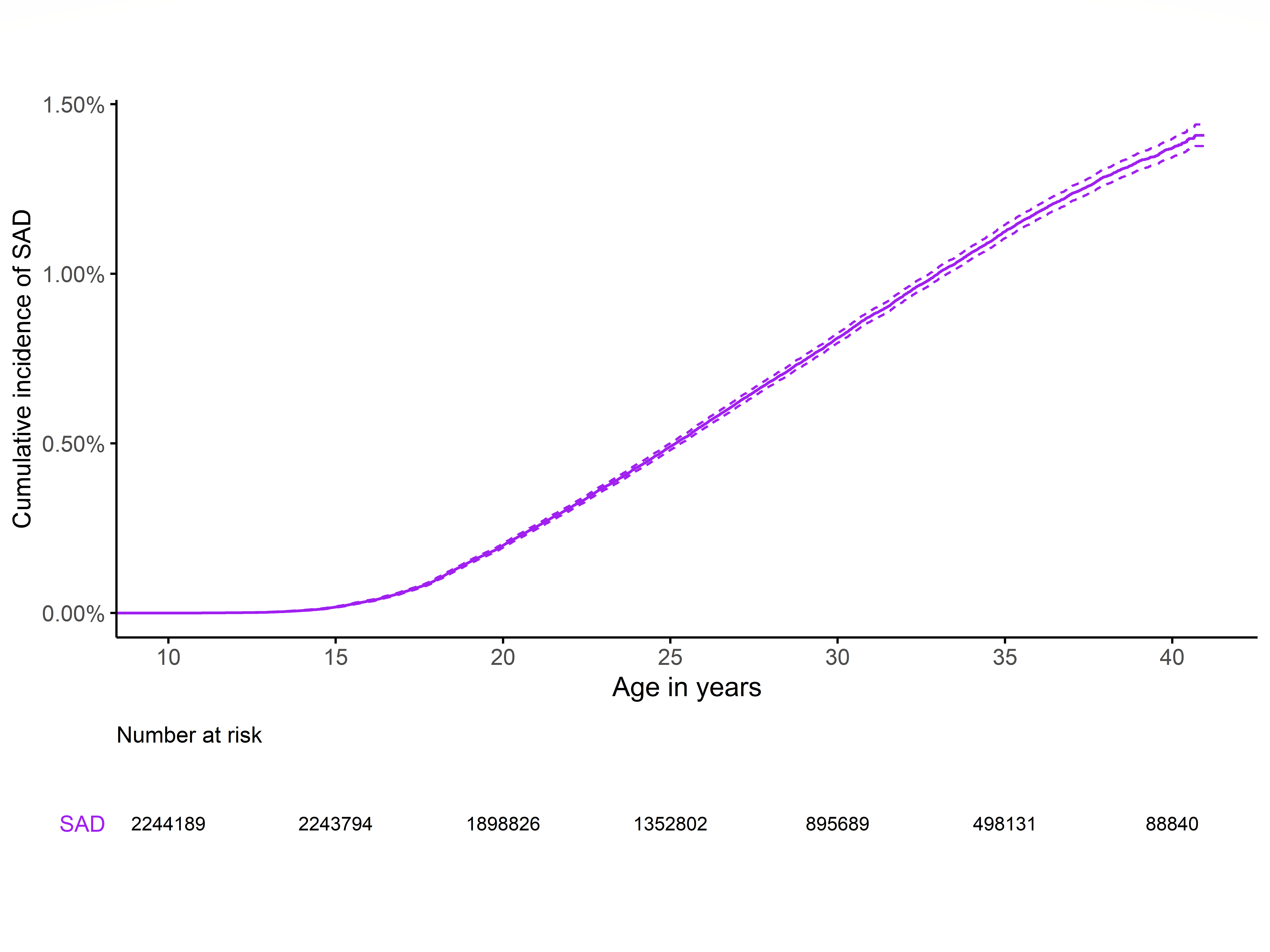

Supplement: Supplementary file 1 [file S0033291719003908sup.zip › S0033291719003908sup001.tif]
